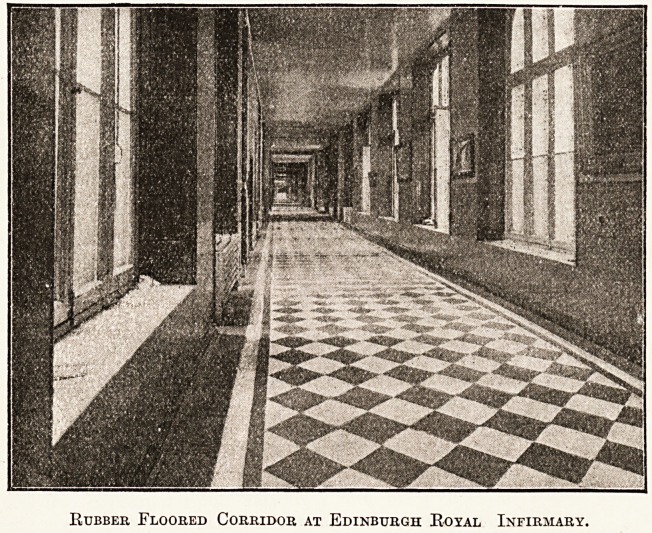# India-Rubber Flooring for Hospital Work

**Published:** 1915-01-23

**Authors:** Frank Ed. B. Blanc

**Affiliations:** Architect.


### INDIA-RUBBER FLOORING FOR HOSPITAL WORK.

By FRANK ED. B. BLANC, Architect.

It is only within recent years that india-rubber has been extensively applied for floor covering, although isolated instances of such application appear as far back as thirty years ago. Within the last decade, however, there are many notable instances where rubber flooring has been effectively and satisfactorily employed in steamships, hotels, insurance offices, banks, and public buildings. Two fine examples of this work, executed about ten years ago, may be cited : the vestibule of the North British Station Hotel in Glasgow and the offices of the North British and Mercantile Insurance Company in Edinburgh. These floors are extremely effective, and show no signs of deterioration or wear.

While no one doubted that rubber flooring would be particularly suitable for hospital buildings or other like institutions, it remained for this to be demonstrated through the generosity of the Rubber Growers' Association, who, in order to popularise the use of the material, have made gifts of rubber floors to the Edinburgh Royal Infirmary, Guy's Hospital, and the Hospital for Sick Children in Great Ormond Street, London.

The use of india-rubber for flooring purposes attracted my attention some years ago, and several of my clients have adopted it in their buildings in place of other coverings. I was therefore specially interested when entrusted with the preparation of the designs for the flooring given to the Royal Infirmary of Edinburgh.

### Æsthetic Adaptability.

So far as the use of the material is concerned, it may be stated that its adaptability to artistic schemes of both design and colour is one of its advantages over other flooring materials. It may confidently be said that it can be applied to a wider range of treatment than any other single form of floor covering. Thus it may be designed to resemble black-and-white marble tiling, or, if desired, made more decorative to imitate a Turkey carpet. Again, an imitation of "old English" terra-cotta tiles can be produced. Even the veining effect of marble has been produced by a recent patent. There are besides effects obtainable by what are known as interlocking rubber tiles. These consist of tiles of two distinct forms, one of which dovetails into the other, and by varying the colours of these very good effects may be produced.

Probably the chief advantage of this form of flooring arises from the physical properties of rubber. In addition to the agreeable elasticity to the tread that rubber naturally possesses, and its thorough cleanliness, its perhaps most valuable quality is the absolute lack of noise. The difference in a large public building laid with any form of flooring compared with one covered with rubber is most noticeable, and this, of course, is a telling point in favour of rubber flooring for hospital work. Again, rubber is non-slipping, and provides a sure foothold.

### The Life of Rubber Floors.

Next to this is the question of durability. On this subject some remarkable points come out on investigation. I am informed that, provided rubber of sufficiently good quality is used, well laid, and not abused in any fashion, there is no reason why the life of rubber flooring should not be practically unlimited. This sounds rather a "tall" order, but evidence is not wanting. As an instance, rubber pavement was laid opposite shop premises in Princes Street, Edinburgh, over which the foot traffic is very considerable. This paving was down for thirty years, and when lifted owing to the removal of the tenants it was found not to have worn 5 per cent. during that long period, whereas the flagstones surrounding it had been renewed several times. Similar evidence comes from the factory where this material was made. The rubber was laid on steps which were traversed by several thousands of operatives four times daily, and when lifted after twenty-four years it showed only slight wear at the extreme edge of the step; on the other hand, the stone steps in the same staircase, which had been left uncovered during that period, had become worn away to the extent of quite two inches.

THE NEW HALL FLOOR AT EDINBURGH ROYAL INFIRMARY.

### A Necessary Warning.

In this connection it is well to add a word of warning, that, if satisfactory results are to be obtained from this material as a form of floor covering, the quality must be good, and it ought to be laid on a thoroughly level surface. "Rubber" is an elastic term, as it is supposed to be an elastic substance; but samples have been submitted to me of so-called rubber tiling which appear to be a compound of putty and paint, which readily broke when bent. It is essential that those who propose to adopt this material should satisfy themselves that it possesses the necessary elasticity, and that it is really rubber, otherwise the results are sure to turn out disappointing. Another point to be careful about is that the quality is inodorous.

Now as to its particular suitability and application to hospital work. In the case of the Edinburgh Royal Infirmary, as will be seen from the illustrations, the entrance hall and corridors extending on either side of it were covered with rubber flooring, the total area being about 800 square yards. Off from these corridors, which are practically the main arteries of the infirmary, and therefore subject to very heavy traffic, branch various wards. The floors hitherto have been stone-flagged, and consequently the noise of the constant traffic was very noticeable and undoubtedly disturbing to patients. The absolute cessation of all noise since the rubber flooring was laid has been most noteworthy, and has had the effect of making the infirmary staff wish it were installed in other parts of the building. The flooring here was manufactured and laid by the North British Rubber Company, Ltd. Great credit is due to them for the way in which they carried out the somewhat complicated design in the main entrance hall. This comprised the incorporation of the infirmary crest, encircled by a border of classic design with many intricate parts.

At Guy's Hospital, I understand, the flooring was laid down the centre of one of the wards, and here again I imagine its quietness will be found an inestimable boon to the patients. The Leyland and Birmingham Rubber Company, Ltd., undertook to carry out the work.

It may be stated that in both these instances the rubber flooring was laid under conditions similar to those existing in other institutions. In each case, however, the form of the gift necessitated that the flooring should, as far as possible, be visible to the public who visited the hospitals, and as a consequence the spaces open to be considered were naturally circumscribed.

Rubber Floored Corridor at Edinburgh Royal Infirmary.

### Rubber Floors for Theatre Work: A Practical Objection.

My view is that, as regards the specialised hospital work more particularly, rubber has a very wide field. For an operating theatre, for instance, it seems to me that it could not well be improved upon. Rubber flooring, giving the effect of black-and-white tiles, in a modern operating theatre, would not only enhance its appearance, but at the same time augment the light by reflection. Such flooring is strictly non-absorbent, and remains unaffected by temperature within ordinary limits. Of course, it further carries with it the very obvious advantages of silence and cleanliness. All these are qualities which, to my mind, make rubber flooring excellent for an operating theatre. There is only one objection to rubber used in this connection, and that is that strong acids, or even disinfectants, if allowed to remain permanently on the rubber, would cause deterioration. But no injury would result if the flooring were cleaned within a reasonable time, and it may be mentioned in passing that the cleaning of rubber flooring is a matter requiring but little time or trouble. In order to assist cleanliness, a small wood fillet, preferably of hard wood, can with advantage be fixed against the wall and on the top of the rubber. Quite a small fillet will serve the purpose, and apart from its obvious advantages, it gives a finished appearance to the flooring.

There also seems to be scope for the use of rubber tiling in lecture theatres, laboratories, and bathrooms. In the latter cases, the flooring falls into harmony with the glazed tiles of the walls and the porcelain and plated fittings, giving the effect of cleanliness and pleasing efficiency.

Though the initial cost of rubber flooring is rather more than that of ordinary flooring materials, its advantages, I think, more than compensate for the extra cost.

## Figures and Tables

**Figure f1:**
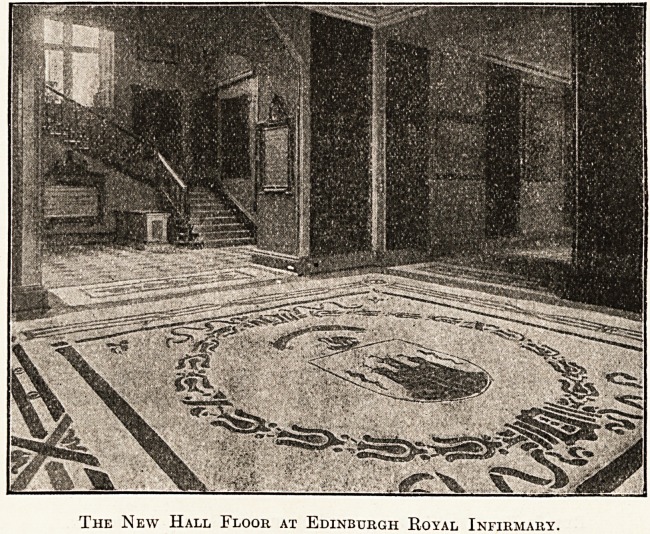


**Figure f2:**